# Lateral chest wall perforator flaps in partial breast reconstruction

**DOI:** 10.1186/s43046-021-00100-5

**Published:** 2022-01-10

**Authors:** Ahmed Orabi, Mina M G Youssef, Tamer M. Manie, Mohamed Shaalan, Tarek Hashem

**Affiliations:** 1 Surgical Oncology Department, National Cancer Institute, Cairo University, Cairo, Egypt; 2 Norfolk and Norwich University Hospital, Norwich, UK; 3 Breast Surgery Department, National Cancer Institute, Cairo University, Cairo, Egypt

**Keywords:** Oncoplastic breast surgery, Volume replacement, LICAP, LTAP, Lateral chest wall perforator flaps

## Abstract

**Background:**

Breast conserving surgery (BCS) has been a standard procedure for the treatment of breast cancer instead of mastectomy whenever possible. Lateral chest wall perforator flaps are one of the volume replacement techniques that participate in increasing the rate of BCS especially in small- to moderate-sized breasts with good cosmetic outcome. In this study, we tried to evaluate the outcome of those flaps as an oncoplastic procedure instead of the conventional flaps.

**Methods:**

This study included 26 patients who underwent partial mastectomy with immediate reconstruction using lateral chest wall perforator flaps in the period from October 2019 to November 2020. The operative time, techniques, and complications were recorded. The cosmetic outcome was assessed 3 months post-radiation therapy through a questionnaire and photographic assessment.

**Results:**

Lateral intercostal artery perforator (LICAP), lateral thoracic artery perforator (LTAP) and combined flaps were performed in 24, 1, and 1 patients, respectively. The mean operative time was 129.6 ± 13.2 min. The flap length ranged from 10 to 20 cm and its width from 5 to 9 cm. Overall patients’ satisfaction was observed to be 88.5% as either excellent or good and the photographic assessment was 96.2% as either excellent or good.

**Conclusions:**

Lateral chest wall perforator flaps are reliable and safe option for partial breast reconstruction with an acceptable aesthetic outcome. In the era of oncoplastic breast surgery, they deserve to gain attention especially with the advantages of some modifications added to the classic technique.

## Background

Perforator flaps are one of the volume replacement oncoplastic techniques that can be used after BCS to reconstruct challenging defects in a relatively large tumor to breast ratio [1]. They are fasciocutaneous flaps that spare muscle function and decrease the morbidity compared to the traditional latissimus dorsi (LD) flap [2]. Lateral chest wall perforator flaps could be based on either lateral thoracic artery or lateral intercostal artery [3]. McCulley et al. described the vascular anatomy and usage of LTAP flap in partial breast reconstruction [3]. The classic LICAP flap technique and the anatomy of lateral intercostal artery perforators were well described by Hamdi et al. [4]. Meybodi et al. added few modifications to overcome the limitations of the classic LICAP flap, avoiding the need to reposition the patient from the supine to the lateral position to harvest the flap. They also shifted the flap design to a more vertical ellipse resulting in a more hidden and better scar [5]. At our institute, we started to offer those flaps to our patients with challenging defects in the breast outer quadrants. The aim of this study is to evaluate the reliability and safety of those flaps in partial breast reconstruction.

## Methods

This is a prospective cohort study that includes 26 patients with early breast cancer who underwent BCS and immediate partial reconstruction with lateral chest wall perforator flaps. Data was recorded from October 2019 to November 2020.

### Inclusion criteria

Patients with T1 or T2 tumors in the outer aspect of the breast where BCS was expected to leave them with a large defect requiring volume replacement with or without skin loss; patients with previous scars of lumpectomy in outer quadrants that needed wider excision and volume replacement; and tumors close to or attached to the skin without infiltration with the flap design modified to recruit skin replacement.

Patients underwent full history, physical examination, standard mammography and ultrasound, core biopsy for diagnosis, contrast-enhanced mammography )CESM(, magnetic resonance imaging (MRI), and staging investigations were performed whenever indicated. Treatment decisions were made by multidisciplinary team (MDT). Routine preoperative blood tests, preoperative flap design marking with the use of handheld Doppler in the lateral or supine position. Intraoperative frozen section was done in all cases for margins assessment.

All operations were carried as a one-night stay in hospital and patients were discharged with the drains in place. Patients were reviewed by the operating surgeon 1 week post-operatively; the drain was removed when collecting less than 50 cc/24 h; and post-operative complications were documented and recorded. Referral to receive adjuvant treatment was done after the final pathology (there was delay in some cases due to COVID-19 pandemic). The patients were followed-up regularly up to at least 6 months following completion of radiotherapy. Cosmetic outcome was evaluated with a modification of the questionnaire of the Royal College of Surgeons of England (3-Month Mastectomy Questionnaire-Royal College of Surgeons www.rcseng.ac.uk› standards-and-research › research) to assess the patients’ reported outcome measures (PROMS). Surgeons’ assessment of post-operative photographs was carried by the operating surgeon and two other experienced oncoplastic surgeons. Their evaluation included volume, shape, symmetry, scar, and nipple-areola complex. Each parameter was scored on a scale from 1 to 5. The total aesthetic outcome score was calculated with a maximum possible score of 25.

### Operative details

**Fig. 1:**
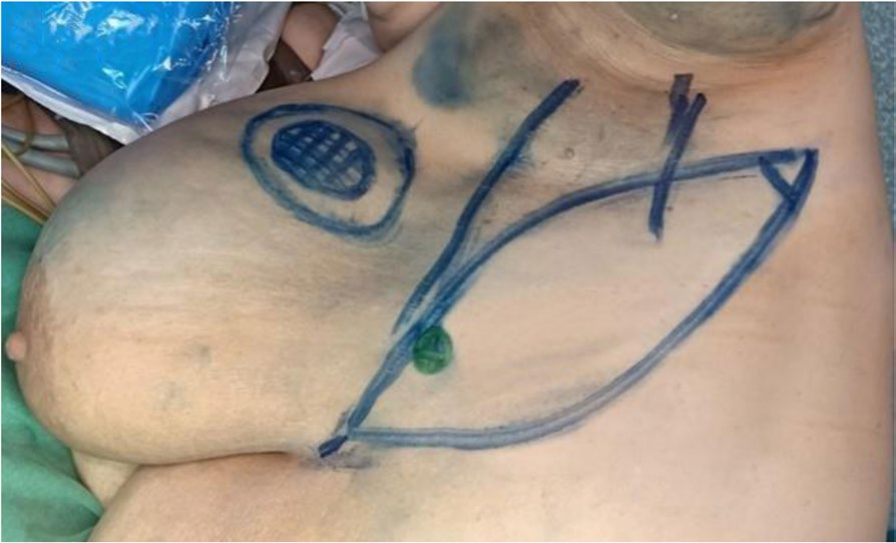
The flap design

Pre-operative flap design was marked in the lateral position. The proximal part of the flap was marked mainly based on the site of the perforators using a handheld Doppler, while the distal part was drawn toward the back. Two elliptical lines were drawn to connect both ends. The width and length of the flap was measured according to the expected defect size following tumor resection (Fig. 1).

The operation was started in the supine position with tumor resection in the standard way from subcutaneous plane to pectoral fascia with handheld monopolar diathermy. The specimen was sent for frozen section to assess the radial resection margins.

**Fig. 2:**
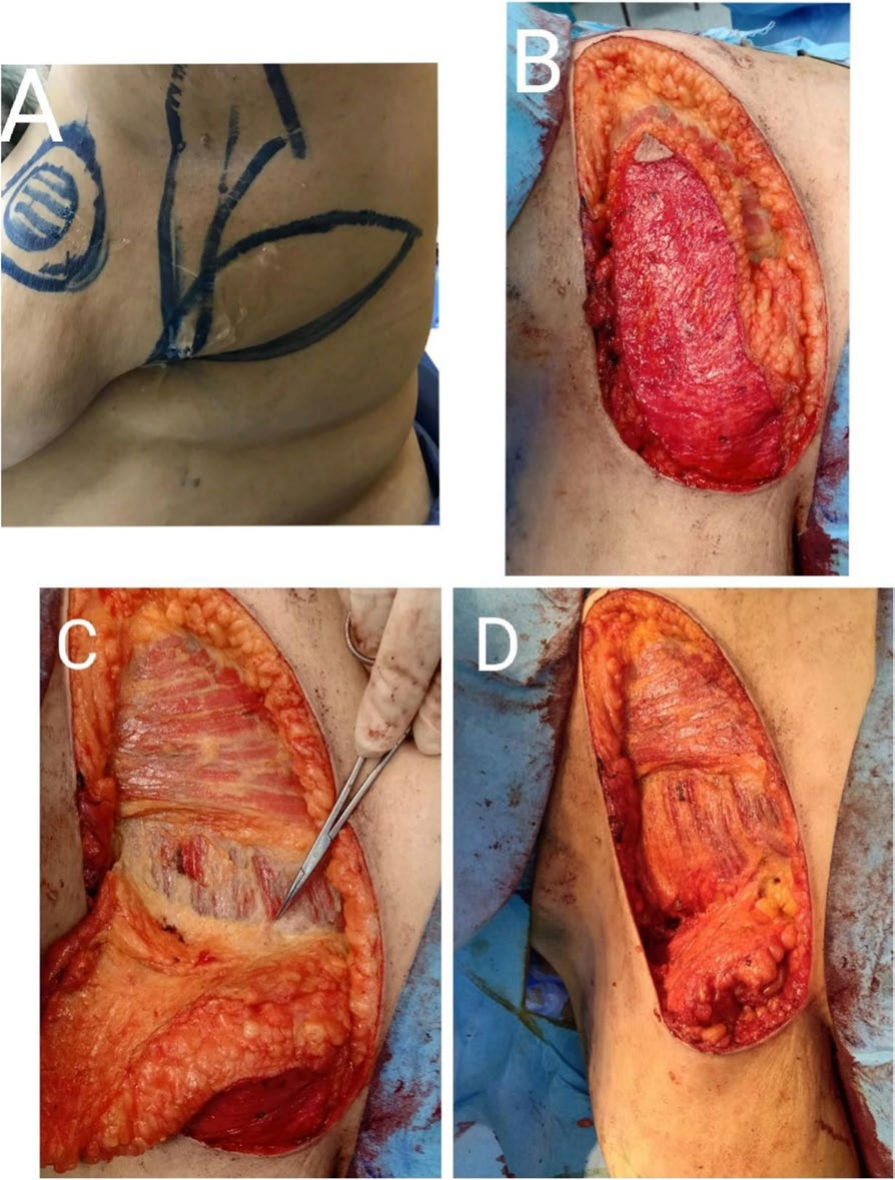
Harvesting of LICAP flap. **A** Preoperative markings of LICAP flap. **B** Starting dissection of the flap form distal side and de-epithelization of the flap skin. **C** Identification of the perforator almost 3 cm anterior to the anterior border of the latissimus dorsi muscle. **D** Rotation of the flap to fill the defect

The patient was then repositioned into the lateral position with a sandbag placed between the chest wall and operating table. The lower arm rested on the procedure table and the upper arm was abducted 90° on an arm board. A pillow was inserted between the legs. Axillary surgery was performed from a separate incision or from the superior border of the flap with care taken to preserve the lateral thoracic artery. After confirming negative margins by frozen section, flap harvesting was initiated from the distal end separating it from the LD muscle, identifying its anterior border until reaching the lateral intercostal artery perforators which were usually located 2 to 3 cm from the anterior border of the LD muscle (in the intercostal spaces from the 5th to the 8th space). We determined the dominant perforator and sacrificed the others that would restrict the mobility of the flap. More dissection and skeletonization of the perforator were done to obtain a longer pedicle. Flap rotation was done to reach the defect and was fixed with vicryl stay sutures (Fig. 2). The patient was repositioned into the supine position and the flap was fixed to the defect with vicryl 2-0 sutures. The donor site was closed primarily with vicryl 2-0 sutures subcutaneously and subcuticular monocryl 3-0 for the skin. This classic technique was done in the initial 3 patients.

In the following 23 patients, we started to modify the technique slightly. While the design and pre-operative marking of the flap remained the same, the procedure was made in the supine position without the need to reposition the patient twice. The skin ellipse design axis was shifted vertically towards the axilla and away from the back. We minimized the skeletonization of the perforator vessels and rather kept the tissues around them acting as a mesentery for additional security.

We used the handheld Doppler to locate the perforators pre-operatively. We used it intra-operatively in the first 5 patients. After we observed almost a constant anatomical location for the perforators, we depended on the Doppler less often in the following 21 patients.

The flap was posted to the defect in two possible ways: the propeller method, was used when skin replacement was required to be resected either (due to tumor attachment or in re-excision cases). The flap was rotated (clockwise or counterclockwise) and posted into the defect. The turnover method was used when skin resection was not required. The incision was made in the antero-superior line of the flap marking. De-epithelization of the skin of the flap was done and the harvested flap was inset by folding it 180° based on the perforator.

In one case, we found that the lateral thoracic artery perforator was the dominant one located in the 4th intercostal space 3 cm anterior to the anterior border of the LD muscle. Here, dissection was started from caudal to cephalad. In another case, we found two good perforators: one belonging to the LTAP and the other to the LICAP. We preserved both to maximize the flap perfusion.

Data was analyzed using IBM SPSS advanced statistics (Statistical Package for Social Sciences), version 24 (SPSS Inc., Chicago, IL). Numeric data was expressed as mean and standard deviation while qualitative data was described as number and percentage.

## Results

### Patients’ characteristics

The total number of patients was 26 with a mean age of 41.12 years (range 22–59). One patient was a type 2 diabetic, and another was hypertensive. None of the patients was a smoker or with ischemic heart disease. The body mass index (BMI) mean was 28.12 kg/m^2^ (range 22–38). Breast cup size was B, C, and D in 15, 9, and 2 cases, respectively.

### Tumor characteristics

There were 2 patients with stage I (7.7%), 22 patients with stage II (84.6%) and 2 patients with stage IIIA (7.7%) due to N2 axilla (T1, N2) and (T2, N2) (Table 1). The mean of T stage was 3.7 ± 0.8 ranged from 2 to 4.5 cm. Nodal status was N1 in 20 patients (77%) and N2 in 2 patients (7.7%). In N2 patients, neoadjuvant chemotherapy was recommended by the MDT to downstage the tumor. Right sided tumors were seen in 14 cases.

**Table 1:** Clinical staging of the patients

Stage	Number of patients	%
I	2	7.7
II	22	84.6
IIIA	2	7.7

The tumor located in the upper outer quadrant (UOQ), lower outer quadrant (LOQ), and upper inner quadrant (UIQ) in 20 (76.9%), 5 (19.2%), and 1 patient (3.8%) respectively. Eight cases received neoadjuvant therapy due to either tumor biology (HER2-enriched or triple negative) or axillary nodal disease.

### Operative data (Table 2)

Twenty patients (76.9%) underwent wide local excision while the other 6 patients (23.1%) were referred to our institute for re-excision to clear the margins after their initial surgery.

**Table 2:** Operative data

		*N* = 26	%
Operation	Wider excision	6	23.1
	Wide local excision	20	76.9
Frozen section		26	100.0
Axillary clearance (AC)	AC	22	84.6
(Range)		(11–30)	
(Mean ± Sd)		16.6 ± 5.1	
Sentinel lymph node (SLN)	SLN	4	15.4
(Range)		(3–5)	
(Mean ± Sd)		3.8 ± 1.0	
Flap	Combined	1	3.8
	LICAP	24	92.3
	LTAP	1	3.8
Method	Propeller	20	76.9
	Turnover	6	23.1
Defect size (length)			
(Range)		(4–7)	
(Mean ± Sd)		5.6 ± 1.1	
Defect size (width)			
(Range)		(7-13)	
(Mean ± Sd)		10.5±1.8	
Flap Size (length) (mean ± Sd)		15.88 ± 2.36	
(Range)		(10-20)	
Flap size (width) (mean ± Sd)		7.35 ± 1.16	
(Range)		(5–9)	
Time (min)		129.6 ± 13.2	
One versus two stages	One	26	100.0
Specimen weight		149.00 ± 29.0	
Pathology	Invasive duct carcinoma	24	92.3
	Medullary	1	3.8
	Mixed	1	3.8
Molecular subtype	Luminal	23	88.5
	HER2-enriched	2	7.7
	Triple negative	1	3.8
Grade	1	1	3.8
	2	19	73.1
	3	6	23.1
Margins	Negative	26	100.0
Complications	No	24	92.3
	Fat Necrosis	1	3.8
	Keloid	1	3.8
Re-excision	No	26	100.0

Mean specimen weight was 149 gm ± 29. Twenty-two patients (84.6%) underwent axillary clearance while 4 patients (15.4%) underwent sentinel lymph node biopsy (SLNB). All patients underwent immediate reconstruction after confirming negative resection margins by intraoperative frozen section. LICAP, LTAP, and combined flaps were used in 24 (92.3%), 1 (3.8%), and 1 patient (3.8%), respectively. The mean operative time was 129.6 ± 13.2 min. The flap length ranged from 10 to 20 cm and its width from 5 to 9 cm. The handheld Doppler was used pre-operatively to locate the perforators in all patients and intra-operatively in 5 cases only )19.2%).

#### Post-operative data

The hospital stay was one day for all patients. The mean of duration for drain removal was 12.4 ± 2.8 ranged from 7 to 15 days. No donor site morbidity was reported. No further surgery was required after the final pathology report. All patients received adjuvant radiotherapy. 24 (92.3%), 18 (69.2%), and 12 patients (46.1%) received hormonal therapy, chemotherapy, and target therapy, respectively. One patient (3.8%) developed keloid and one (3.8%) developed fat necrosis diagnosed clinically and radiologically with MRI. No patient needed contralateral symmetrization.

**Fig. 3:**
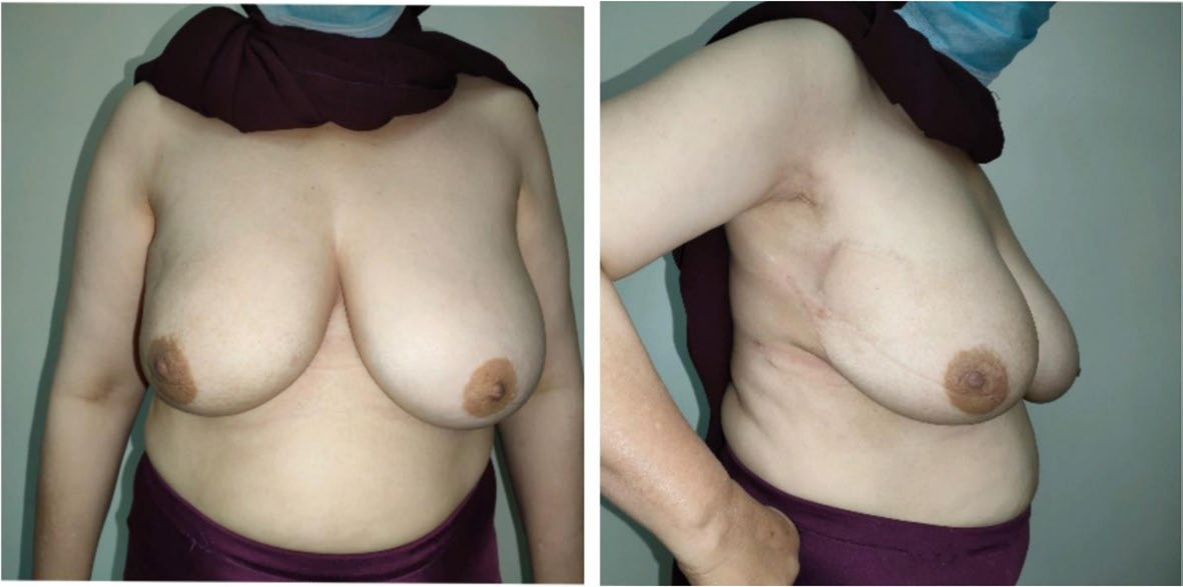
A 49-year-old female patient with right LICAP 9 months post-radiation therapy

**Fig. 4:**
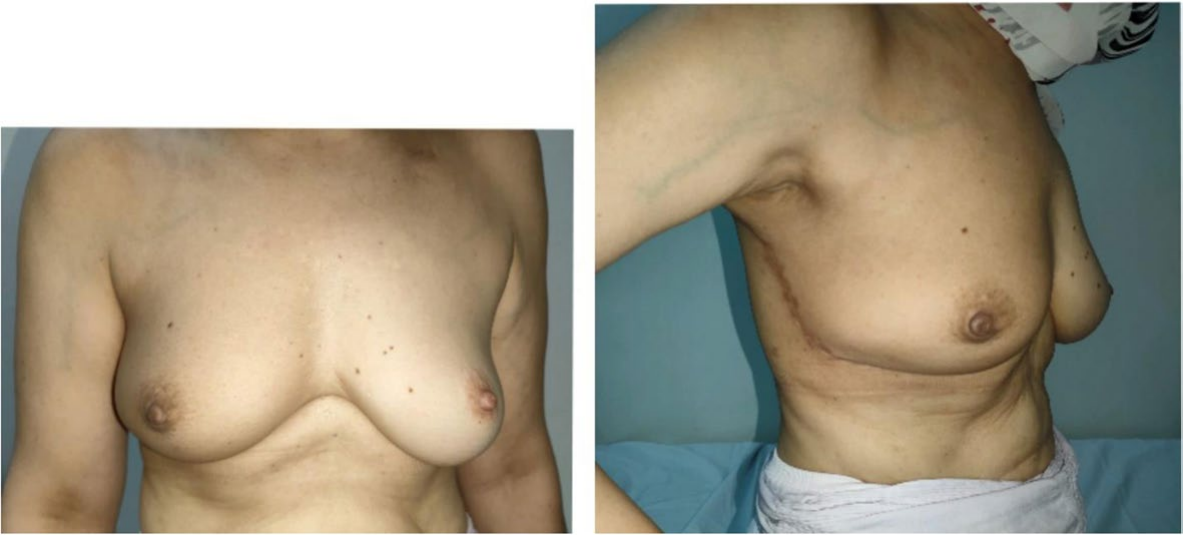
A 59-year-old female patient with right LICAP 12 months post-radiation therapy

Patients’ satisfaction reported in the questionnaire was excellent (65.4%), good (23.1%), and fair (11.5%) (Table 3). Cosmetic outcome assessment by surgeons was excellent (65.4%), good (30.8%), and fair (3.8%) (Table 4) (Figs. 3 and 4).

**Table 3:** Patient’s satisfaction results

Excellent	Good	Fair	Poor	Very poor
65.4%	23.1%	11.5%	Nil	Nil

**Table 4:** The photographic assessment results

Excellent	Good	Fair	Poor	Very poor
65.4 %	30.8 %	3.8 %	Nil	Nil

Two patients in our cohort were offered contralateral reduction mammoplasty for symmetry but declined.

## Discussion

The use of intercostal artery perforator to supply a musculocutaneous flap has been described since the 1970s [6]. LICAP was introduced by Hamdi et al. as a fasciocutaneous flap in reconstruction of lateral breast defects after partial mastectomy with the advantage of sparing the LD, preserving its function and its use as a flap in cases of local recurrence [7]. Several authors reported successful outcomes and advantages of the LICAP flap in challenging lateral breast defects [1, 8, 9]. Meybodi et al. published their modifications to the traditional LICAP flap, reporting a safer and faster technique with a better scar cosmetically [5].

We describe in this cohort the first series of this technique at our institute. The consultant surgeons involved in this study were exposed to such technique in different overseas centers which allowed them to transfer the experience to the junior surgeons locally.

Hamdi et al. described the classic technique for LICAP flap [4]. In our study, we started by performing the classic technique in the first three patients (11.5%), then we introduced few modifications as our learning curve progressed in the subsequent patients (88.5%). We found that this technique particularly time-saving mainly by eliminating the need to reposition the patient. The other noted advantage was the superior cosmetic outcome associated with a vertical lateral scar that allows better access to the axilla at the same time.

Our mean operative time was 129.6 ± 13.2 min compared to 249.3 ± 40.1 min as reported by Kim et al. [8].

The majority of authors reported using handled Doppler to locate and mark the perforator pre-operatively [3, 4, 8], and sometimes intra-operatively [9]. In our study, we used the handheld doppler to mark the perforator pre-operatively. As we progressed with the learning curve, the intra-operative confirmation of their constant anatomical position depended less on the use of Doppler. This study highlighted the almost constant anatomical location of lateral intercostal artery perforators. We observed that perforators in all cases located in a triangle between the lateral mammary fold, the inframammary fold, and the anterior axillary line. The confirmation of the perforators’ position depended less on the intra-operative Doppler as the team built more experience.

Some series reported repeat surgery for wider excision to clear the margins [10] or for axillary clearance due to positive SLNB [5]. In our institute, the availability of intraoperative frozen section eliminated the need for re-operation allowing a safe immediate reconstruction.

The reported complications in the literature include flap venous congestion [3, 8], partial flap necrosis [11, 10], wound infection [5, 11], fat necrosis [8, 10], hematoma [10], and seroma [10]; in our cohort, there were no reported immediate postoperative complications. Two patients (7.7%) developed late complications, in the form of keloid and fat necrosis and were managed conservatively without surgical intervention.

One patient was referred to our tertiary center with a scar extending to the UIQ following a lumpectomy with infiltrated margins in a small- to medium-sized breast. She underwent a wider excision, leaving a defect that extended to the UIQ. It was possible to extend the flap to cover this defect safely. This could be a promising option for reconstruction of defects in the UIQ in selected cases. Further studies are needed to evaluate this possible expansion of the technique.

LTAP flap is reported in the literature for the reconstruction of lateral breast defects either alone or combined with LICAP to maximize the flap perfusion [3]. In our study, we used LTAP flap in one case in which the defect was in the UOQ, where the lateral thoracic artery perforator was dominant and reliable. In another patient, we performed it combined with the LICAP in which both perforators were prominent.

We used a modification of the questionnaire of the Royal College of Surgeons of England as a simple tool to assess patients' satisfaction which was (88.5%) as excellent and good, and the photographic assessment by surgeons which was (96.2 %) as excellent and good. The results were matching with those reported by Kim et al, they use Kyungpook National University Hospital Breast Satisfaction Survey (93.1% and 93%) by patients and surgeons respectively [8].

Lateral chest wall perforator flaps initially described for small-sized breasts [9]. This indication expanded to medium- and large-sized breasts in further series [5, 10]. In our cohort, all patients were medium to large-sized breasts.

Regarding modifications to the flap design, we used the lazy S shaped incision toward the axilla described by Meybodi et al. Recently, Juliëtte et al. described the anterior LICAP flap with a different flap design toward the inframammary fold [12].

We recognize many limitations to our study. One main limitation is the small number of patients in the cohort and the relatively short time of follow-up. This reflects the reality of a newly adopted technique at our institute, the follow-up period extended up to at least 6 months after completion of radiotherapy and the patients continue to be followed-up further. We used a simple tool for assessment of cosmetic outcomes, as we felt it was more suitable for our patient population. Follow-up will be continued to assess long term cosmetic outcomes in patients with medium- to large-sized breasts by using other cosmetic results assessment tools.

## Conclusions

The LICAP flap is a safe and reliable option for reconstruction of large challenging defects in the lateral aspect of the breast. Those perforator vessels have a constant anatomical position and therefore the flap has a relatively easy learning curve. The modifications described in this cohort simplified the technique further leading to a shorter operating time. The post-operative complications were minimal with high patient satisfaction even after radiotherapy. In this study, we did not aim to focus on specific outcome of the LTAP flap. Therefore, its reliability in partial breast reconstruction needs further studies for detailed evaluation.

## Data Availability

The raw data and materials are available upon request.

## Abbreviations

AC: Axillary clearance
BCS: Breast conserving surgery
BMI: Body mass index
CESM: Contrast enhanced mammography
LD: Latissimus dorsi
LICAP: Lateral intercostal artery perforator
LOQ: Lower outer quadrant
LTAP: Lateral thoracic artery perforator
MDT: Multidisciplinary team
MRI: Magnetic resonance imaging
PROMS: Patients’ reported outcome measures
SLNB: Sentinel lymph node biopsy
UIQ: Upper inner quadrant
UOQ: Upper outer quadrant

## References

[1] Munhoz AM, Montag E, Arruda E, Pellarin L, Filassi JR, Piato JR, et al. Assessment of immediate conservative breast surgery reconstruction: a classification system of defects revisited and an algorithm for selecting the appropriate technique. Plast Reconstr Surg. 2008;121(3):716e27. 18317121 10.1097/01.prs.0000299295.74100.fa

[2] Hamdi M, Van Landuyt K, Monstrey S, Blondeel P. Pedicled perforator flaps in breast reconstruction: a new concept. Br J Plast Surg. 2004;57(6):531e9. 15308400 10.1016/j.bjps.2004.04.015

[3] McCulley SJ, Schaverien MV, Tan VK, Macmillan RD. Lateral thoracic artery perforator (LTAP) flap in partial breast reconstruction. J Plast Reconstr Aesthet Surg. 2015;68(5):686–91. 25660559 10.1016/j.bjps.2015.01.008

[4] Hamdi M, Van Landuyt K, de Frene B, Roche N, Blondeel P, Monstrey S. The versatility of the inter-costal artery perforator (ICAP) flaps. J Plast Reconstr Aesthet Surg. 2006;59(6):644–52. 16716957 10.1016/j.bjps.2006.01.006

[5] Meybodi F, Cocco AM, Messer D, Brown A, Kanesalingam K, Elder E, et al. The modified lateral intercostal artery perforator flap. Plast Reconstr Surg Glob Open. 2019;7(2):e2066. 30881825 10.1097/GOX.0000000000002066 PMC6416140

[6] Badran HA, El-Helaly MS, Safe I. The lateral intercostal neurovascular free flap. Plast Reconstr Surg. 1984;73:17e23. 6361828 10.1097/00006534-198401000-00003

[7] Hamdi M, Spano A, Van Landuyt K, D'Herde K, Blondeel P, Monstrey S. The lateral intercostal artery perforators: anatomical study and clinical application in breast surgery. Plast Reconstr Surg. 2008;121(2):389e96. 18300954 10.1097/01.prs.0000298317.65296.cf

[8] Kim JB, Eom JR, Lee JW, Lee J, Park HY, Yang JD. Utility of two surgical techniques using a lateral intercostal artery perforator flap after breast-conserving surgery: a single-center retrospective study. Plast Reconstr Surg. 2019;143(3):477e–87e. 10.1097/PRS.0000000000005374 30817638

[9] Schwartz JD. New approach to oncoplastic breast conservation: combining autologous volume replacement and the wise-pattern mammaplasty. Plast Reconstr Surg Glob Open. 2018;6(10):e1987. 30534509 10.1097/GOX.0000000000001987 PMC6250486

[10] Roy PG. One-stage vs. two-stage approach for partial breast reconstruction with lateral chest wall perforator flaps. Cancer Treat Res. 2016;9:56–61.

[11] Hakakian CS, Lockhart RA, Kulber DA, Aronowitz JA. Lateral intercostal artery perforator flap in breast reconstruction: a simplified pedicle permits an expanded role. Ann Plast Surg. 2016;76(Suppl 3):S184–90. 26914351 10.1097/SAP.0000000000000752

[12] Jacobs JED, Al Shaer S, Schmidbauer U, de Leeuw DM, Rakhorst HA, Zöphel OT. The anterior LICAP flap: a design option for oncoplastic breast reconstruction. Case Reports Plast Surg Hand Surg. 2021;8(1):158–63. 34621915 10.1080/23320885.2021.1986048 PMC8491666

